# Skeletal muscle index and hand grip strength as predictors of postoperative recovery in patients with gastrointestinal tumors

**DOI:** 10.1186/s12876-026-04850-1

**Published:** 2026-04-23

**Authors:** Mehdi Azizmohammad Looha, Zahra Sharifi, Sepideh Banar, Seyedali Vakily, Mehdi Eshaghzadeh, Amirhassan Rabbani

**Affiliations:** 1 Basic and Molecular Epidemiology of Gastrointestinal Disorders Research Center, Research Institute for Gastroenterology and Liver Diseases, Shahid Beheshti University of Medical Sciences, Tehran, Iran; 2 Faculty of Medicine, Iran University of Medical Sciences, Tehran, Iran; 3 Department of Cardiothoracic Surgery, Erasmus University Medical Centre, Rotterdam, The Netherlands; 4 Department of Surgery, Shahid Beheshti University of Medical Sciences, Tehran, Iran; 5 Department of Radiology, Ayatollah Taleghani Hospital, Shahid Beheshti University of Medical Sciences, Tehran, Iran; 6 Department of Surgery, Ayatollah Taleghani Hospital, Shahid Beheshti University of Medical Sciences, Tehran, Iran

**Keywords:** Sarcopenia, Gastrointestinal Neoplasms, Muscle, Skeletal, Hand Strength, Postoperative complications

## Abstract

**Background:**

Sarcopenia, defined by loss of muscle mass and function, predicts poor cancer outcomes. In gastrointestinal (GI) malignancies, its prognostic role remains underexplored. Skeletal muscle index (SMI) from computed tomography (CT) scans and hand grip strength (HGS) may contribute to preoperative risk assessment. This study aimed primarily to evaluate the association between preoperative SMI and postoperative hospitalization length, and secondarily to assess HGS as a functional measure related to muscle status and its association with SMI and other clinical factors in GI cancer patients.

**Methods:**

A prospective observational cohort study was conducted (2019–2022) at a tertiary hospital in Tehran. Patients with GI malignancies undergoing surgery and preoperative CT scans were included. SMI was measured at the L3 vertebral level using standardized thresholds. The primary outcome was hospitalization length; HGS was secondary. Negative binomial regression was used to evaluate predictors of hospitalization length, and linear regression models were used to examine factors associated with HGS. Univariate and multivariable analyses were performed.

**Results:**

A total of 233 patients were included in the study. The mean age was 59.13 ± 12.74 years, and 56.7% were male. Higher SMI was associated with shorter hospitalization length in both univariate and multivariable analyses (Incidence rate ratios [IRRs] = 0.97 and 0.94, respectively; *P* < 0.001). HGS was not associated with hospitalization length. Psoas muscle diameter showed a small positive association with hospitalization length in the multivariable model (IRR = 1.01; *P* = 0.032). SMI was positively associated with HGS in univariate analyses for both hands (right hand: β = 0.31; *P* = 0.027; left hand: β = 0.32; *P* = 0.016), but this association was not retained after adjustment. Age and psoas muscle diameter remained independent predictors of HGS in both hands.

**Conclusion:**

Preoperative SMI appears to be a clinically relevant imaging-based marker of postoperative recovery in patients with GI cancers and may contribute to preoperative risk assessment. HGS, evaluated as a functional measure of muscle status, was associated with SMI and selected clinical factors but did not provide comparable prognostic information for hospitalization outcomes. These findings support the complementary but distinct roles of structural (SMI) and functional (HGS) muscle assessments. Further studies are required to validate these findings and clarify their clinical application.

**Supplementary Information:**

The online version contains supplementary material available at 10.1186/s12876-026-04850-1.

## Introduction

Gastrointestinal (GI) cancers are highly prevalent and contribute substantially to cancer-related mortality [1]. Despite advances in surgical and oncological treatments, postoperative complications remain a major challenge [2–4]. While factors such as age, obesity, and physical fitness provide some prognostic value [3, 4], there is increasing interest in objective and quantifiable markers to better assess physiological reserve and recovery potential [5, 6].

Sarcopenia, the progressive loss of skeletal muscle mass and function, is an independent predictor of adverse outcomes in cancer patients, including increased postoperative complications, prolonged hospitalization, and reduced survival [7–9]. Traditional measures such as body mass index (BMI), which do not distinguish between muscle and fat, have limited value in oncological settings [10]. Therefore, the assessment of muscle strength and sarcopenia provides a more accurate evaluation of physical status. Influenced by age, malnutrition, and comorbidities, sarcopenia significantly affects prognosis, highlighting the importance of its early detection and management [11–13].

While dual-energy X-ray absorptiometry (DEXA) is the gold standard for assessing muscle mass [14], computed tomography (CT) is more commonly used in clinical practice. Skeletal muscle index (SMI), calculated from the total skeletal muscle cross-sectional area at the level of the third lumbar vertebra (L3) on CT scans, is widely recognized as a reliable proxy for muscle depletion and sarcopenia [15]. Reduced SMI has been associated with poorer surgical outcomes, supporting its role in preoperative risk assessment [16–18]. In addition to muscle mass, functional status is also a key determinant of outcome prediction. Hand grip strength (HGS) is a simple measure of muscle strength and nutritional status, and lower HGS has been linked to higher cancer mortality and poorer preoperative status [19–23]. Further evidence supports the importance of muscle strength in sarcopenic populations [15, 24].

Sarcopenia is an important risk factor for adverse postoperative outcomes in GI malignancies [25]. It is prevalent in gastric cancer and is associated with increased complications, including severe and respiratory events after gastrectomy [25–27], and with poorer outcomes and delayed recovery in colorectal cancer [28–30]. As a potentially modifiable condition, early identification may improve surgical prognosis [31–33]. However, most evidence is derived from non-Iranian populations, particularly lacking data from Middle Eastern settings, highlighting the need for region-specific studies.

In this context, identifying reliable and clinically applicable markers of muscle status may improve preoperative prognostic assessment and guide targeted interventions. The primary aim of this study was to evaluate the association between preoperative SMI and postoperative hospitalization length in patients with GI tumors. As a secondary objective, HGS was assessed as a functional measure of muscle status to characterize its relationship with SMI and to determine whether it reflects a similar underlying dimension of muscle condition. To better understand the determinants of HGS and clarify whether its variation reflects intrinsic muscle status or is influenced by broader clinical factors, we additionally examined its associations with key clinical variables, including age and BMI. Given the practical limitations of SMI assessment, including reliance on imaging, cost, and technical expertise, we further evaluated whether HGS may serve as a complementary and clinically accessible bedside measure, rather than a substitute for imaging-based muscle assessment.

## Materials and methods

### Study design and setting

This study was designed as a prospective observational cohort study conducted at Ayatollah Taleghani Hospital, affiliated with Shahid Beheshti University of Medical Sciences, Tehran, Iran. The study period spanned from 2019 to 2022. The research adhered to the ethical principles outlined in the Declaration of Helsinki, and ethical approval was obtained from the Ethics Committee of Shahid Beheshti University of Medical Sciences (IR.SBMU.MSP.REC.1401.376). All participants provided written informed consent prior to enrollment, and all data were de-identified before analysis. Participants who declined to provide consent were not enrolled. This study was reported in accordance with the Strengthening the Reporting of Observational Studies in Epidemiology (STROBE) guidelines.

### Participants

Patients diagnosed with GI malignancies, including gastric, colon, hepatic, and gallbladder cancers, were eligible for inclusion. Only patients who underwent surgical treatment and had available preoperative abdominopelvic CT imaging were included in the study cohort. Missing data occurred primarily at the variable level rather than through complete exclusion of patients; therefore, all eligible participants were retained in the overall cohort and contributed to analyses for which the required variables were available. Thus, analysis-specific available-case or complete-case approaches were applied depending on the requirements of each analysis. For clarity, missing data were handled using an analysis-specific complete-case approach, whereby participants were included in each analysis only if all required variables were available. In particular, analyses requiring simultaneous availability of multiple variables were conducted using a complete-case approach, resulting in a reduced sample size for those specific analyses. Missingness was mainly attributable to practical and technical constraints in data availability and processing, rather than predefined exclusion criteria. A separate variable distinguishing elective from emergency surgery was not consistently available in the dataset; therefore, eligibility was not defined on that basis. Instead, differences in operative context were addressed analytically through surgical procedure category and surgical complexity, as described below.

### Variables

#### Outcome variables

The primary outcome of this study was the length of postoperative hospital stay, measured in calendar days from the date of surgery to the date of discharge. This variable was used to assess postoperative recovery and was recorded by trained clinical staff.

The secondary outcome was HGS, reflecting muscle status rather than a primary clinical outcome. HGS was assessed using a JAMAR Plus+ digital hand dynamometer (Patterson Medical, Warrenville, IL, USA), which was calibrated according to the manufacturer’s instructions before each measurement session. The JAMAR dynamometer is widely recognized as the gold standard for HGS assessment and has demonstrated excellent validity, as well as high intra- and inter-rater and test–retest reliability across both healthy and clinical populations [34–37].

Assessments were performed with patients seated comfortably, with the shoulder adducted and neutrally rotated, the elbow flexed at 90 degrees, the forearm in a neutral position, and the wrist positioned between 0 and 30 degrees of dorsiflexion. Grip strength was measured in both the right and left hands, and each hand was tested three times with a 30-second rest interval between attempts. The highest value (in kilograms) for each hand was recorded and used in the analysis. All HGS measurements were performed by trained assessors following a standardized protocol. Inter-rater reliability was formally evaluated using the intraclass correlation coefficient (ICC), demonstrating excellent agreement (ICC = 0.92). Intra-rater reliability was not assessed due to the absence of repeated measurements by the same assessor. Both hands were analyzed separately, and no restriction to dominant or non-dominant hand was applied.

#### Demographic variables

Patient characteristics that define the study population included age, sex, height, and weight. These variables were recorded at the time of enrollment and provided baseline information for the analysis.

#### Confounding variables

Potential confounders that could influence the relationship between skeletal muscle index and clinical outcomes included BMI, cancer type, surgical procedure category, and comorbidities. In addition, surgical procedure category was incorporated as a clinically important confounder, and surgical complexity was defined to reflect the expected operative burden. Surgical approach (minimally invasive vs. open) was reviewed but was not included in the final adjusted models because, after complete-case restriction, it showed insufficient variability and incomplete documentation for stable estimation.

To improve the assessment of comorbidity burden and address potential limitations of using a simple comorbidity count, a modified Charlson-like comorbidity score was constructed. Because a standardized Charlson Comorbidity Index (CCI) was not directly available in the dataset, comorbid conditions were extracted from free-text clinical records and categorized into clinically relevant groups using predefined keyword-based identification.

Major comorbid conditions including diabetes mellitus, cardiovascular disease (including coronary artery disease, ischemic heart disease, myocardial infarction, and prior revascularization procedures), cerebrovascular disease, chronic liver disease, chronic kidney disease, chronic pulmonary disease, and malignancy were identified and weighted according to a simplified Charlson-style scoring system. Each condition was assigned a weight based on its clinical severity and established relevance to postoperative outcomes, with higher weights assigned to malignancy and chronic kidney disease. The resulting composite score (Charlson-like score) was used as a covariate in regression analyses. Additionally, the score was categorized into clinically interpretable groups (0, 1, 2, and ≥ 3) to facilitate analysis and interpretation. Because this modified score was derived from available data rather than a validated instrument, it should be interpreted as an approximate measure of comorbidity burden.

Surgical procedures were classified into broad procedure categories for adjustment in the regression models, rather than being grouped solely by anatomical listing. Specifically, procedures were categorized as hepatopancreatobiliary procedures, upper gastrointestinal procedures, gastrointestinal procedures, general and emergency procedures, and a small residual category requiring manual review. Because an explicit elective-versus-emergency designation was not consistently recorded, the ‘general and emergency procedures’ category and the separately defined surgical complexity variable were used to partially capture differences in operative context and expected perioperative burden. Hepatopancreatobiliary procedures included hepatectomy, partial hepatectomy, liver lobectomy, liver transplantation, resection of hepatic cyst or mass, cholecystectomy, excision of choledochal cyst, distal pancreatectomy, distal subtotal pancreatectomy, and the Whipple procedure. Upper gastrointestinal procedures included esophagectomy, esophagectomy via left thoracotomy, and gastrectomy-related procedures. Gastrointestinal procedures encompassed colectomy (partial, total, hemicolectomy, sigmoidectomy), proctectomy, proctocolectomy, and polypectomy for colon polyps. Procedures that did not fit clearly within these main groups were placed in the “other/manual review” category after independent clinical re-evaluation. Because procedures within the same anatomical category may still differ substantially in physiological burden and expected postoperative recovery (e.g., minor procedures versus highly complex oncologic resections), surgical procedure category was used primarily as a broad clinical adjustment variable. To better account for operative burden and reduce residual confounding arising from this heterogeneity, surgical complexity was defined separately as low, intermediate, or high, and considered as a complementary adjustment variable in sensitivity analyses.

### SMI measurement

Preoperative abdominopelvic CT scans were acquired using a Aquilion 16-slice CT scanner (Toshiba Medical Systems, Japan) without intravascular contrast injection. Imaging parameters were standardized across patients and included a tube voltage of 120 kVp and a tube current ranging from 200 to 250 mA, with automatic exposure control applied where available. Images were reconstructed at a slice thickness of 5 mm using a standard soft-tissue reconstruction algorithm (kernel) optimized for abdominal imaging. Axial images at the level of L3 were used for all muscle measurements. Skeletal muscle segmentation was performed using a semi-automated approach within the Slice-O-Matic software to quantify the total skeletal muscle cross-sectional area at the level of L3, including the psoas, paraspinal, and abdominal wall muscles. Muscle tissue was identified using predefined Hounsfield unit (HU) thresholds (− 29 to + 150 HU), which are widely validated for CT-based skeletal muscle assessment in sarcopenia research. All segmentations were independently performed and reviewed by two blinded physicians. Inter-rater reliability was assessed using the ICC, demonstrating excellent agreement between observers (ICC = 0.94). The SMI was then calculated using the following formula:

$$\:{S}{M}{I}=\frac{{\text{Total skeletal muscle area at L3 (cm}}^{2}\mathrm{)}}{{\text{Height (m)}}^{2}}$$

In the primary analyses, SMI was treated as a continuous variable and used directly in regression models without categorization. To address potential ambiguity related to different sarcopenia definitions, categorical SMI variables were constructed only for sensitivity analyses using established CT-based thresholds. Specifically, main sarcopenia was defined based on the International Cancer Cachexia Consensus criteria as follows: men: SMI < 55 cm²/m² and women: SMI < 39 cm²/m² (primary definition); and an alternative definition using lower cut-offs (men: < 49 cm²/m²; women: < 31 cm²/m²) [38]. These categorical variables were not used in the primary models but were included in supplementary analyses to evaluate the robustness of findings across different threshold definitions.

**Fig. 1:**
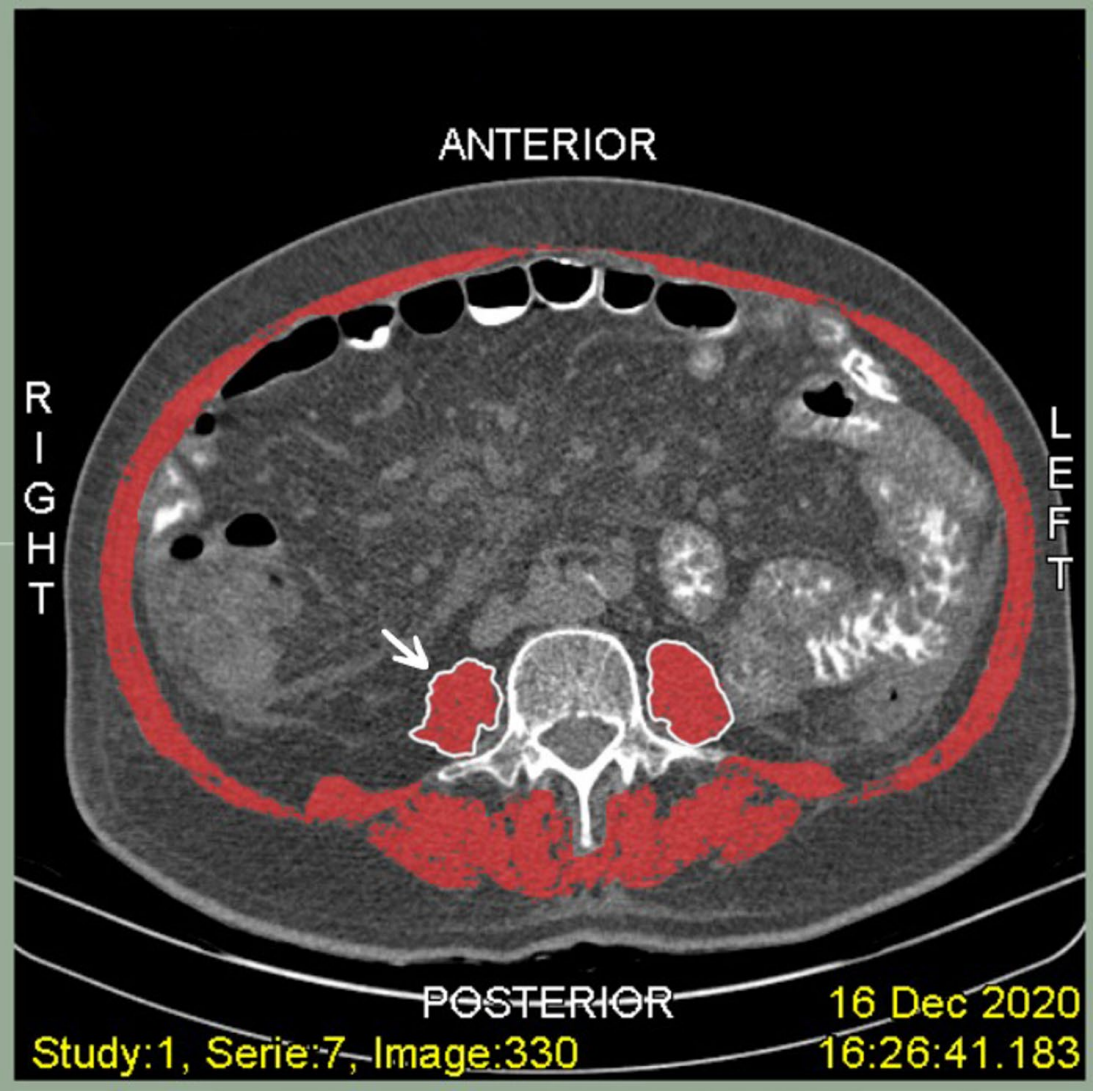
Representative axial CT image at the level of L3 demonstrating skeletal muscle segmentation for calculation of SMI. The highlighted regions (in red) represent the total skeletal muscle area, including the psoas, paraspinal, and abdominal wall muscles, identified using standardized Hounsfield unit (HU) thresholds for muscle tissue (− 29 to + 150 HU). Segmentation was performed using a semi-automated approach and subsequently verified by two independent blinded assessors. The psoas muscles are shown bilaterally adjacent to the vertebral body for anatomical reference

There is currently no universally accepted threshold for defining sarcopenia. Therefore, SMI was primarily analyzed as a continuous variable to avoid arbitrary categorization and preserve statistical power. Following the image acquisition process, representative CT scan images were selected to illustrate the method used for SMI measurement (Fig. 1). The figure demonstrates segmentation of skeletal muscle at the L3 level based on standardized HU thresholds and reflects the applied semi-automated segmentation process.

Missing data were primarily related to incomplete availability of imaging-derived or functional measurements rather than systematic exclusion, and were therefore handled using analysis-specific complete-case approaches.

### Study size

The required sample size for this study was determined using the non-central F distribution method for multiple linear regression, which does not have a closed-form equation and is therefore computed iteratively. To obtain an accurate estimate, we used the pwr.f2.test() function from the pwr R package [39], and verified the result using G*Power 3.1 software [40]. This method is based on the following steps and formulas:

1. Non-centrality parameter:

$$\mathrm{lambda}\;=\mathrm{f}^2\;\ast\;\text{(v + u + 1)}$$

2. Critical F value (based on alpha):

$$\mathrm{F}_\mathrm{crit}\;=\;\mathrm{F}_{\_1\;-\;\mathrm{alpha}}\mathrm{(u,v)}$$

(i.e., the (1 - alpha)-quantile of the central F-distribution with u and v degrees of freedom)

3. Power function (non-central F distribution):

$$\mathrm{Power}=\;1 -\;\mathrm{P}(\mathrm{F}_{\text{u, v, lambda}}\;\leq\;\mathrm{F}_{\mathrm{crit}})$$

4. Final required sample size:

$$\mathrm{n}\;=\mathrm{v}\;+\;\mathrm{u}\;+1$$

In this analysis, f² = R² / (1 - R²) represents Cohen’s effect size, and we used f² = 0.22 (expected effect size of skeletal muscle index). The number of independent predictors was u = 5, and v denotes the degrees of freedom for the residuals (which was not assumed in advance, but determined iteratively within the power analysis process to satisfy the desired power level), which was determined iteratively to meet the desired statistical power. The total required sample size is calculated as n = v + u + 1. Using this method, the required sample size to achieve 80% power at a significance level of 0.05 was calculated to be *n* = 65.

### Statistical analysis

First, descriptive statistics were used to summarize the study variables. Continuous variables were presented as mean ± standard deviation (SD) or median (interquartile range [IQR]), as appropriate based on distribution, while categorical variables were reported as frequencies (percentages). Missing data were handled using analysis-specific approaches, with available-case methods applied for descriptive analyses and complete-case approaches used for multivariable models.

Second, to evaluate predictors of hospitalization length, univariate Poisson regression models were initially fitted for each independent variable. Over-dispersion was formally assessed using both the ratio of Pearson chi-square to residual degrees of freedom and the dispersion test implemented in the AER package in R. Given clear evidence of over-dispersion, negative binomial (NB) regression models were subsequently used for both univariate and multivariable analyses. For the multivariable analysis of hospitalization length, a screened approach was applied in which variables with *P* < 0.10 in univariate NB analyses were considered for inclusion. This screening-based modeling strategy was consistently applied across all multivariable analyses of hospitalization length, including those presented in the Supplementary Materials. For categorical predictors, if at least one level met this threshold, the entire variable was retained in the model. Model adequacy and goodness-of-fit were evaluated using log-likelihood, Akaike information criterion (AIC), and Bayesian information criterion (BIC). Multicollinearity was assessed using variance inflation factors (VIFs). For categorical variables, adjusted generalized variance inflation factors (GVIF^(1/(2×Df))) were used.

Third, to address potential confounding related to surgical heterogeneity, surgical procedure category was included as a baseline adjustment variable, and surgical complexity was additionally evaluated to better capture operative burden. Because a direct elective-versus-emergency variable was not consistently available, surgical complexity was also used as a proxy for operative urgency. A sensitivity analysis was conducted by incorporating surgical complexity (low, intermediate, high) into the screened multivariable NB model, to further account for potential confounding by surgical procedure. Due to complete-case restriction, the low-complexity category was not represented in the multivariable dataset; therefore, surgical complexity was modeled as a binary variable (high vs. intermediate). The results of this sensitivity analysis were compared with those of the corresponding screened multivariable model to assess robustness. Surgical approach (minimally invasive vs. open) was evaluated as a potential covariate but was not included in the final models due to insufficient completeness and variability.

Fourth, for the analysis of HGS, univariate linear regression models were first performed for both right and left hands separately. Because HGS was analyzed as a secondary functional outcome, multivariable linear regression models were constructed using a forward stepwise selection approach. SMI, as the prespecified primary exposure, was forced into all multivariable models regardless of statistical significance to ensure estimation of its adjusted association with HGS. Cancer type was also included as a covariate in all multivariable models to account for clinical heterogeneity. In addition, stratified analyses were performed across predefined cancer subgroups (gastrointestinal, hepatobiliary, and pancreatic/periampullary malignancies). Sensitivity analyses were conducted to compare models using continuous SMI with those using categorical sarcopenia definitions based on established CT-derived thresholds.

Fifth, to explore unadjusted relationships between key variables, Spearman’s rank correlation analysis was performed to assess pairwise associations between SMI, hospitalization length, age, BMI, and HGS. These analyses were considered exploratory and descriptive, as they do not account for confounding, and therefore were not used for causal or independent inference. Potential confounding was addressed separately using multivariable regression models. Correlation analyses were conducted using complete-case datasets, resulting in reduced sample sizes depending on variable availability.

Analysis populations were explicitly defined according to the availability of variables required for each model. For the primary outcome (hospitalization length), patients with available outcome data were included, while multivariable models were restricted to complete-case datasets requiring simultaneous availability of all covariates. Similarly, analyses of HGS and correlation analyses were performed using complete-case datasets specific to each model. As a result, effective sample sizes varied across analyses. To evaluate the potential impact of missing data and assess the risk of selection bias, baseline characteristics of patients included in complete-case analyses were compared with those excluded. Given that missingness was primarily driven by structural and technical constraints rather than outcome-dependent mechanisms, complete-case analysis was considered the most transparent and appropriate approach, although the possibility of selection bias cannot be fully excluded.

## Results

### Baseline clinical and demographic characteristics

A total of 233 patients were included in the final study cohort. During the study period, 240 patients were initially identified, of whom 7 were excluded due to absence of usable study data, resulting in the final analytic cohort of 233 patients. However, the number of patients contributing to each analysis varied according to variable availability, as detailed in Table 1 and further illustrated in the study flow diagram presented in Supplementary Figure S1. The mean age of participants was 59.13 ± 12.74 years, and the majority were male (56.65%). The average BMI was 24.88 ± 5.28 kg/m². The most common cancer type was gastrointestinal malignancies (40.34%), followed by hepatobiliary malignancies (30.47%) and pancreatic and periampullary malignancies (29.18%).

**Table 1:** Baseline characteristics of the study population (*n* = 233)

Variable	Level / Unit	Value	*N* (Non-missing)	Missing
Demographic variables				
Sex	Female	101 (43.35%)	233	0
	Male	132 (56.65%)	233	0
Age (years)	—	59.13 ± 12.74	232	1
Anthropometric and functional variables				
BMI (kg/m²)	—	24.88 ± 5.28	170	63
Psoas muscle diameter (mm)	—	122.34 ± 27.39	120	113
HGS – Right (kg)	Total	25.12 ± 8.04	169	64
	Male	28.13 ± 7.75	103	29
	Female	20.43 ± 6.00	66	35
HGS – Left (kg)	Total	23.76 ± 8.12	169	64
	Male	26.88 ± 8.06	103	29
	Female	18.87 ± 5.39	66	35
SMI (cm²/m²)	—	45.54 ± 8.04	74	159
Comorbidity variables				
Number of comorbidities (continuous)	—	0.78 ± 1.10	233	0
Number of comorbidities (categorical)	0	127 (54.51%)	233	0
	1	56 (24.03%)	233	0
	2	27 (11.59%)	233	0
	≥ 3	23 (9.87%)	233	0
Modified Charlson-like score	—	0.42 ± 0.73	233	0
Charlson-like category	0	161 (69.7%)	231	2
	1	52 (22.5%)	231	2
	2	18 (7.8%)	231	2
	≥ 3	2 (0.86%)	231	2
Cancer characteristics				
Cancer type	Gastrointestinal malignancies	94 (40.34%)	233	0
	Hepatobiliary malignancies	71 (30.47%)	233	0
	Pancreatic & periampullary malignancies	68 (29.18%)	233	0
Surgical characteristics				
Surgical procedure category	Hepatopancreatobiliary procedures	132 (56.65%)	233	0
	Upper gastrointestinal procedures	46 (19.74%)	233	0
	Gastrointestinal procedures	40 (17.17%)	233	0
	General & emergency procedures	8 (3.43%)	233	0
	Other / review manually	7 (3.00%)	233	0
Surgical complexity	Low	17 (7.30%)	233	0
	Intermediate	70 (30.04%)	233	0
	High	146 (62.66%)	233	0
Outcome variable				
Hospitalization length (days)	Total (Mean ± SD)	6.30 ± 3.32	136	97
	Total (Median, IQR)	6 (4–8)	136	97
	Low complexity (Mean ± SD)	8.50 ± 5.07	4	13
	Low complexity (Median, IQR)	7.50 (4.25–13.75)	4	13
	Intermediate complexity (Mean ± SD)	5.78 ± 2.94	37	33
	Intermediate complexity (Median, IQR)	5.00 (4.00–7.00)	37	33
	High complexity (Mean ± SD)	6.41 ± 3.37	95	51
	High complexity (Median, IQR)	6.00 (4.00–8.00)	95	51

Continuous variables are presented as mean ± standard deviation (SD), except for hospitalization length, which is reported as both mean ± SD and median (interquartile range, IQR) due to its skewed distribution. Categorical variables are expressed as frequency (percentage). The number of non-missing and missing observations is reported for each variable to ensure transparency in data completeness. Comorbidity burden was assessed using both the unweighted number of comorbid conditions and a modified Charlson-like comorbidity score derived from available clinical data. Surgical procedures were categorized based on anatomical and clinical classification, and surgical complexity was defined as low, intermediate, or high according to procedural extent and expected physiological burden. Hospitalization length was additionally stratified by surgical complexity to improve interpretability of postoperative recovery patterns across different levels of operative burden. Surgical approach was reviewed as a clinically relevant variable but was not included in the final adjusted models because the available complete-case data showed insufficient variability and incomplete documentation. Skeletal Muscle Index (SMI) was calculated based on the total skeletal muscle cross-sectional area at the level of the third lumbar vertebra (L3), including the psoas, paraspinal, and abdominal wall muscles, normalized for height squared (cm²/m²), and should not be interpreted as a psoas-only measurement. A separate elective-versus-emergency surgery variable was not consistently available; operative context was therefore represented indirectly by surgical procedure category and surgical complexity.

*Abbreviations*: *BMI* body mass index, *HGS* hand grip strength, *SMI* skeletal muscle index, *IQR* interquartile range

### Psoas diameter and SMI (total muscle area-based)

The mean psoas muscle diameter was 122.34 ± 27.39 mm. SMI had a mean value of 45.54 ± 8.04 cm²/m² and was available for 74 patients.

### Hospitalization duration and HGS measurements

The overall mean hospitalization length was 6.30 ± 3.32 days, with a median of 6 days (IQR: 4–8), based on 136 patients with available outcome data. When stratified by surgical complexity, the mean hospitalization length was 8.50 ± 5.07 days in the low-complexity group, 5.78 ± 2.94 days in the intermediate-complexity group, and 6.41 ± 3.37 days in the high-complexity group; the corresponding medians (IQRs) were 7.50 (4.25–13.75), 5.00 (4.00–7.00), and 6.00 (4.00–8.00), respectively.

HGS was measured for both hands. Measurements were available for 169 patients in each hand. The overall mean HGS was 25.12 ± 8.04 kg for the right hand and 23.76 ± 8.12 kg for the left hand. In sex-stratified analyses, mean right-hand HGS was 28.13 ± 7.75 kg in males and 20.43 ± 6.00 kg in females, while mean left-hand HGS was 26.88 ± 8.06 kg in males and 18.87 ± 5.39 kg in females.

### Factors affecting hospitalization length

As presented in Table 2, hospitalization length was analyzed using NB regression due to evidence of over-dispersion in Poisson models. In univariate NB analyses, SMI was significantly inversely associated with hospitalization length, with each 1 cm²/m² increase corresponding to a 3% reduction in the expected duration of hospital stay (incidence rate ratios [IRR] = 0.97; 95% CI: 0.95–0.98; *P* < 0.001). Cancer type showed a partially significant association, as patients with pancreatic and periampullary malignancies had a significantly higher hospitalization rate compared with those with hepatobiliary malignancies (IRR = 1.37; 95% CI: 1.08–1.74; *P* = 0.009), while the difference between gastrointestinal and hepatobiliary malignancies was not statistically significant. Patients with a Charlson-like comorbidity category of 1 (indicating the presence of a single comorbid condition) also demonstrated a significantly higher hospitalization rate compared to those without comorbidities (IRR = 1.27; 95% CI: 1.04–1.54; *P* = 0.018). In addition, several surgical procedure categories were associated with significantly lower hospitalization rates compared with the reference category (general and emergency procedures), including upper gastrointestinal procedures (IRR = 0.41; 95% CI: 0.17–0.92; *P* = 0.032), gastrointestinal surgical procedures (IRR = 0.43; 95% CI: 0.18–0.98; *P* = 0.048), hepatopancreatobiliary procedures (IRR = 0.42; 95% CI: 0.18–0.92; *P* = 0.034), and procedures classified as “other/manual review” (IRR = 0.36; 95% CI: 0.13–0.96; *P* = 0.044). Other variables were not significantly associated with hospitalization length in univariate analyses.

**Table 2:** Univariate and multivariable NB models for predictors of hospitalization length

Variable	Comparison / Unit	Univariate NB			Screened Multivariate NB		
		IRR (95% CI)	P-value	AIC/BIC	IRR (95% CI)	P-value	AIC/BIC
Age	Per 1-year increase	1.00 (0.99, 1.00)	0.364	699.64 / 708.38	—	—	332.48 / 354.07
BMI	Per 1-unit increase	1.00 (0.98, 1.02)	0.689	535.72 / 543.65	—	—	
Sex	Male vs. Female	1.05 (0.88, 1.25)	0.585	700.15 / 708.89	—	—	
Cancer type	Pancreatic and periampullary malignancies vs. hepatobiliary malignancies	1.37 (1.08, 1.74)	0.009	695.80 / 707.45	1.21 (0.84, 1.76)	0.306	
	Gastrointestinal malignancies vs. hepatobiliary malignancies	1.19 (0.95, 1.48)	0.133		1.01 (0.54, 1.97)	0.974	
Psoas muscle diameter	Per 1-unit increase	1.00 (0.99, 1.00)	0.086	464.58 / 472.01	1.01 (1.00, 1.02)	0.032	
HGS – Right	Per 1-kg increase	1.00 (0.99, 1.02)	0.690	531.19 / 539.06	—	—	
HGS – Left	Per 1-kg increase	1.00 (0.99, 1.02)	0.786	531.27 / 539.14	—	—	
Charlson-like category	1 vs. 0	1.27 (1.04, 1.54)	0.018	696.70 / 711.26	1.25 (0.98, 1.58)	0.069	
	2 vs. 0	0.91 (0.65, 1.26)	0.579		1.15 (0.75, 1.70)	0.513	
	≥ 3 vs. 0	0.50 (0.11, 1.64)	0.294		—*	—*	
Surgical procedure category	Upper gastrointestinal procedures vs. general and emergency procedures	0.41 (0.17, 0.92)	0.032	701.40 / 718.87	—†	—†	
	Gastrointestinal surgical procedures vs. general and emergency procedures	0.43 (0.18, 0.98)	0.048		1.04 (0.77, 1.39)	0.814	
	Hepatopancreatobiliary procedures vs. general and emergency procedures	0.42 (0.18, 0.92)	0.034		0.88 (0.43, 1.88)	0.738	
	Other / review manually vs. general and emergency procedures	0.36 (0.13, 0.96)	0.044		—†	—†	
SMI	Per 1 cm²/m² increase	0.97 (0.95, 0.98)	< 0.001	329.05 / 335.53	0.94 (0.92, 0.97)	< 0.001	

Incidence rate ratios (IRRs) and 95% confidence intervals (CIs) were estimated using negative binomial regression models. Univariate analyses were conducted separately for each predictor. The screened multivariable negative binomial model included predictors that demonstrated evidence of association in the univariate negative binomial analyses at a threshold of *P* < 0.10; for categorical variables, once a predictor met this criterion, all of its levels were retained in the model. Reference categories were defined as female for sex, hepatobiliary malignancies for cancer type, Charlson-like category 0 for comorbidity burden, and general and emergency procedures for surgical procedure category. The ≥ 3 level of the Charlson-like category (*) was not estimated in the screened multivariable model, likely due to sparse data or instability after complete-case restriction. Additionally, some surgical procedure contrasts (†) were not displayed in the final model output, which may reflect their role as reference categories, reparameterization during model fitting, or non-estimability within the reduced complete-case dataset. Skeletal Muscle Index (SMI) represents total skeletal muscle area at the L3 level and is distinct from psoas muscle measurements, which were included as a separate covariate. Multicollinearity was assessed using variance inflation factors (VIF) and adjusted generalized VIF (GVIF^(1/(2×Df))), with all values below 5, indicating no evidence of problematic collinearity

*Abbreviations*: *IRR* incidence rate ratio, *CI* confidence interval, *BMI* body mass index, *HGS* hand grip strength, *NB* negative binomial, *SMI* skeletal muscle index

In the screened multivariable NB model, SMI remained a significant independent predictor of hospitalization length (IRR = 0.94; 95% CI: 0.92–0.97; *P* < 0.001), corresponding to an approximate 6% reduction in expected hospitalization duration per unit increase in SMI. Psoas muscle diameter also showed a small but statistically significant positive association with hospitalization length (IRR = 1.01; 95% CI: 1.00–1.02; *P* = 0.032). The effect of Charlson-like comorbidity category (1 vs. 0) was attenuated and did not remain statistically significant after adjustment (*P* = 0.069). Cancer type and surgical procedure categories were retained in the model based on the predefined screening criteria; however, their adjusted associations were not statistically significant.

### Sensitivity analyses of hospitalization length models

Sensitivity analyses were performed to evaluate the robustness of the associations observed in the screened multivariable model presented in "Factors Affecting Hospitalization Length" section. The results of these analyses are provided in Supplementary Tables S1 and S2. Using alternative exposure definitions, SMI remained significantly associated with hospitalization length when modeled as a continuous variable (adjusted IRR = 0.94; 95% CI: 0.92–0.97; *P* < 0.001) and when defined using the main sarcopenia cut-off (IRR = 1.35; 95% CI: 1.07–1.71; *P* = 0.014) and alternative cut-off (IRR = 1.44; 95% CI: 1.14–1.82; *P* = 0.003). In additional models adjusting for surgical complexity (high vs. intermediate), the effect of SMI remained unchanged (IRR = 0.94; 95% CI: 0.91–0.97; *P* < 0.001), and other covariates showed minimal variation. The low-complexity category was not estimable due to zero observations in the complete-case multivariable dataset. Surgical approach was reviewed but could not be reliably incorporated into the adjusted models because of insufficient variability and incomplete usable data after complete-case restriction.

### Factors affecting HGS

As presented in Table 3, univariate linear regression analyses showed that age, sex, psoas muscle diameter, and SMI were significantly associated with HGS in both hands. Increasing age was negatively associated with HGS (right hand: β = -0.29, 95% CI: -0.45 to -0.14, *P* < 0.001; left hand: β = -0.26, 95% CI: -0.41 to -0.11, *P* = 0.001). Male sex was associated with higher HGS compared to females (right hand: β = 7.50, 95% CI: 3.91 to 11.09, *P* < 0.001; left hand: β = 7.37, 95% CI: 3.90 to 10.85, *P* < 0.001). Psoas muscle diameter demonstrated a positive association with HGS in both hands (right hand: β = 0.18, 95% CI: 0.11 to 0.26, *P* < 0.001; left hand: β = 0.18, 95% CI: 0.11 to 0.25, *P* < 0.001). Higher SMI was also significantly associated with higher HGS (right hand: β = 0.31, 95% CI: 0.04 to 0.58, *P* = 0.027; left hand: β = 0.32, 95% CI: 0.06 to 0.58, *P* = 0.016). Other variables were not significantly associated with HGS in univariate analyses.

**Table 3:** Univariate and multivariable linear regression analyses of factors associated with HGS

Dependent Variable	Predictor	Univariate		Multivariate	
		β (95% CI)	P-value	β (95% CI)	P-value
HGS – Right Hand	Age	-0.29 (-0.45, -0.14)	< 0.001	-0.25 (-0.38, -0.12)	< 0.001
	BMI	-0.03 (-0.38, 0.32)	0.858	Not retained	—
	Male vs. Female	7.50 (3.91, 11.09)	< 0.001	3.74 (-0.46, 7.94)	0.080
	Cancer type (Pancreatic vs. Hepatobiliary)	0.29 (-5.49, 6.06)	0.921	Not retained	—
	Cancer type (Gastrointestinal vs. Hepatobiliary)	2.52 (-2.59, 7.62)	0.328	Not retained	—
	Psoas muscle diameter	0.18 (0.11, 0.26)	< 0.001	0.19 (0.03, 0.36)	0.021
	Charlson-like category (1 vs. 0)	-3.96 (-8.33, 0.41)	0.075	Not retained	—
	Charlson-like category (2 vs. 0)	0.13 (-8.12, 8.38)	0.975	Not retained	—
	Surgical procedure (Upper GI vs. General/Emergency)	-5.50 (-22.08, 11.08)	0.509	Not retained	—
	Surgical procedure (GI vs. General/Emergency)	-4.86 (-21.59, 11.87)	0.563	Not retained	—
	Surgical procedure (HPB vs. General/Emergency)	-7.18 (-23.61, 9.26)	0.386	Not retained	—
	Surgical procedure (Other vs. General/Emergency)	-12.90 (-35.76, 9.96)	0.263	Not retained	—
	SMI (per 1 cm²/m² increase)	0.31 (0.04, 0.58)	0.027	-0.28 (-0.72, 0.16)	0.211
HGS – Left Hand	Age	-0.26 (-0.41, -0.11)	0.001	-0.22 (-0.34, -0.09)	0.002
	BMI	0.06 (-0.28, 0.41)	0.718	0.22 (-0.07, 0.51)	0.129
	Male vs. Female	7.37 (3.90, 10.85)	< 0.001	4.65 (0.43, 8.86)	0.031
	Cancer type (Pancreatic vs. Hepatobiliary)	-0.32 (-5.97, 5.33)	0.910	Not retained	—
	Cancer type (Gastrointestinal vs. Hepatobiliary)	1.27 (-3.73, 6.26)	0.614	Not retained	—
	Psoas muscle diameter	0.18 (0.11, 0.25)	< 0.001	0.18 (0.02, 0.34)	0.032
	Charlson-like category (1 vs. 0)	-3.27 (-7.55, 1.00)	0.131	Not retained	—
	Charlson-like category (2 vs. 0)	1.25 (-6.82, 9.31)	0.759	Not retained	—
	Surgical procedure (Upper GI vs. General/Emergency)	-6.30 (-22.45, 9.85)	0.438	Not retained	—
	Surgical procedure (GI vs. General/Emergency)	-6.83 (-23.12, 9.46)	0.405	Not retained	—
	Surgical procedure (HPB vs. General/Emergency)	-7.49 (-23.50, 8.52)	0.353	Not retained	—
	Surgical procedure (Other vs. General/Emergency)	-14.80 (-37.06, 7.46)	0.188	Not retained	—
	SMI (per 1 cm²/m² increase)	0.32 (0.06, 0.58)	0.016	-0.29 (-0.75, 0.17)	0.219

β coefficients and 95% confidence intervals (CIs) were derived from univariate linear regression analyses and multivariable models constructed using a forward stepwise approach. All clinically relevant variables, including cancer type and surgical procedure, were initially evaluated in univariate analyses. Variables were retained in the multivariable models based on statistical and model selection criteria, while skeletal muscle index (SMI) was forced into the models regardless of statistical significance. This was done because SMI was the main prespecified exposure variable and the primary imaging-based marker of muscle mass in the study. Cancer type and surgical procedure were not retained in the final multivariable models due to lack of contribution to model fit. Reference categories were female for sex, hepatobiliary malignancies for cancer type, and general and emergency procedures for surgical procedure. SMI was derived from total skeletal muscle area at the L3 level and should be interpreted as a global muscle mass measure rather than a psoas-specific metric. Multicollinearity was assessed using variance inflation factors (VIF) and adjusted generalized VIF (GVIF^(1/(2×Df))), with all values below 5, indicating no evidence of problematic collinearity

*Abbreviations*: *HGS* hand grip strength, *SMI* skeletal muscle index, *BMI* body mass index, *HPB* hepatopancreatobiliary, *GI* gastrointestinal, *CI* confidence interval

In the multivariable models, age and psoas muscle diameter remained significant independent predictors of HGS in both hands. For the right hand, age remained negatively associated with HGS (β = -0.25, 95% CI: -0.38 to -0.12, *P* < 0.001), while psoas muscle diameter retained a positive association (β = 0.19, 95% CI: 0.03 to 0.36, *P* = 0.021). For the left hand, age (β = -0.22, 95% CI: -0.34 to -0.09, *P* = 0.002) and psoas muscle diameter (β = 0.18, 95% CI: 0.02 to 0.34, *P* = 0.032) remained significant. The effect of sex was attenuated after adjustment and did not remain statistically significant for the right hand (*P* = 0.080), although it remained significant for the left hand (β = 4.65, 95% CI: 0.43 to 8.86, *P* = 0.031). SMI did not retain statistical significance in the adjusted models for either hand (right hand: *P* = 0.211; left hand: *P* = 0.219).

### Correlation network of demographic characteristics, muscle mass, functional strength, and clinical outcomes

**Fig. 2:**
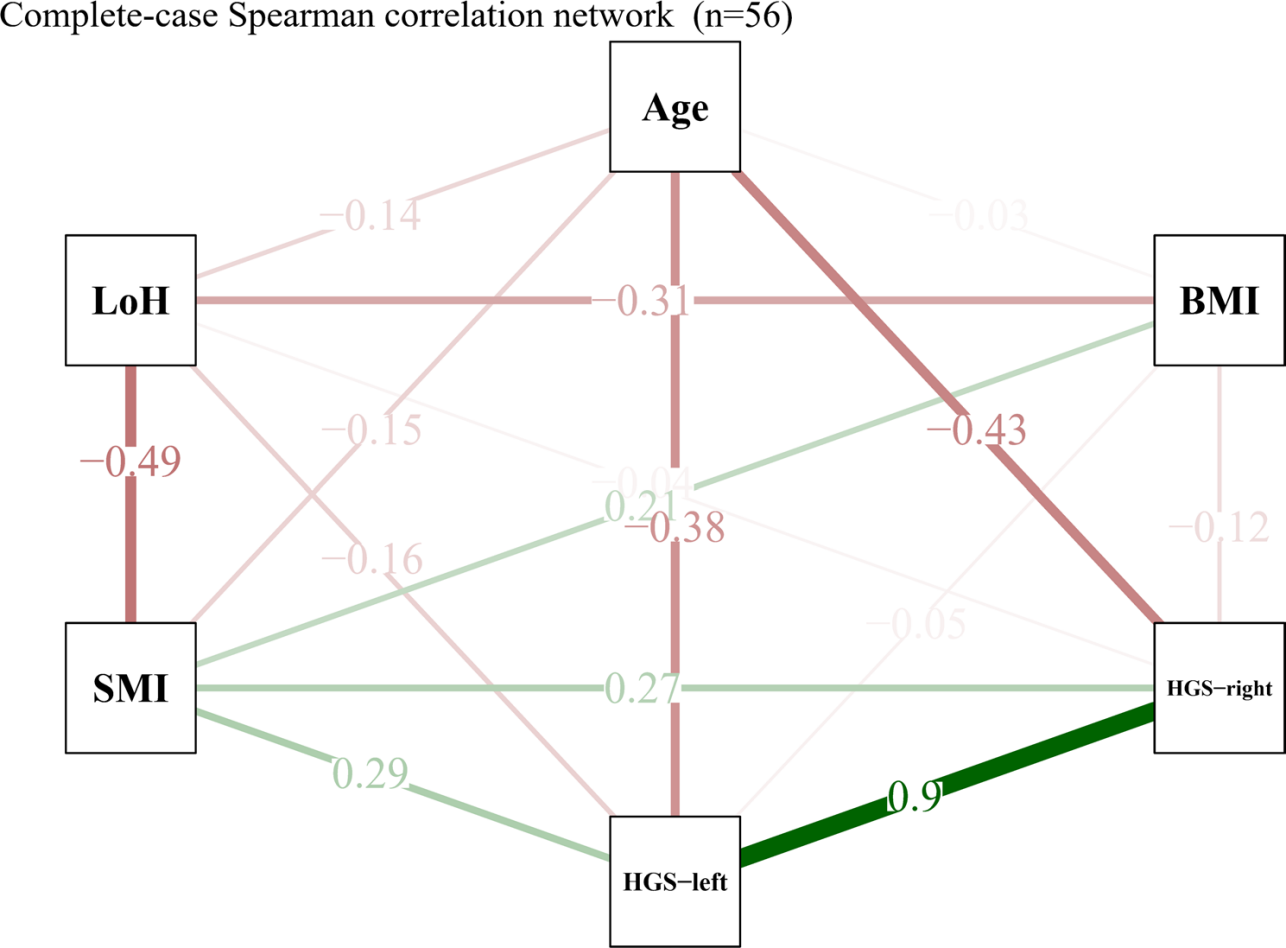
Complete-case Spearman correlation network of SMI, functional variables, and clinical variables. The network diagram illustrates the Spearman correlation coefficients between skeletal muscle index (SMI), hospitalization length (LoH), age, body mass index (BMI), and hand grip strength (HGS) measured in both hands (right and left), based on 56 complete-case observations. Numerical values displayed on each edge represent the corresponding correlation coefficients. The thickness of the edges reflects the magnitude of the correlations, with thicker lines indicating stronger associations. The edge color indicates the direction of the correlation, with green representing positive correlations and red representing negative correlations

Correlation analyses were conducted using a complete-case approach due to missing data in key variables, resulting in a reduced sample size (*n* = 56) compared with the full cohort (*n* = 233). The missing data were primarily related to incomplete availability of imaging-derived measurements (e.g., SMI and psoas muscle diameter) and functional assessments (HGS), rather than systematic exclusion. As shown in Fig. 2 and Supplementary Table S3, Spearman’s correlation analysis demonstrated a significant negative correlation between SMI and hospitalization length (rs = -0.49, *P* < 0.001), as well as between BMI and hospitalization length (rs = -0.31, *P* = 0.021). SMI was positively correlated with HGS in both hands (right hand: rs = 0.27, *P* = 0.047; left hand: rs = 0.29, *P* = 0.033), and a strong positive correlation was observed between right and left HGS (rs = 0.90, *P* < 0.001), while no significant correlation was found between SMI and BMI or age. The correlation network visualization confirmed these relationships, with edge thickness representing the strength of correlations and color indicating direction (green for positive and red for negative correlations), based on the complete-case dataset; the overall pattern of associations remained stable despite the reduced sample size.

### Analysis populations and missing data

Due to variable availability and the use of complete-case approaches in multivariable analyses, the effective sample size differed across statistical models. The screened multivariable model for hospitalization length and the multivariable HGS models each included 64 patients, while the correlation analysis was based on 56 complete cases. Missing data were primarily related to incomplete availability of imaging-derived measurements (e.g., SMI and psoas muscle diameter) and functional assessments (HGS), rather than systematic exclusion of patients at the cohort level. To assess the potential impact of missing data, baseline characteristics of patients included in the correlation analysis were compared with those excluded, as presented in Supplementary Tables S4 and S5, showing broadly similar distributions across key demographic and clinical variables and suggesting that substantial selection bias is unlikely to have materially influenced the observed correlations. A comprehensive summary of analysis-specific sample sizes, variable availability, and variable-level missingness across models is provided in Supplementary Table S6.

### Bootstrap-based assessment of model stability

Bootstrap resampling (1,000 iterations; 566 valid) confirmed the stability of key predictors. SMI showed a consistent negative effect (mean coefficient − 0.029; Exp = 0.971), corresponding to a reduction in hospitalization rate, with high directional consistency (88.2%). Age also demonstrated high stability (mean − 0.012; Exp = 0.988; 93.8% direction consistency). Other covariates exhibited greater variability and lower directional consistency. Detailed results are presented in Supplementary Table S7.

## Discussion

This study demonstrated that preoperative SMI is an independent predictor of postoperative hospitalization length in patients with GI malignancies, with lower SMI consistently associated with prolonged hospital stay after adjustment for relevant clinical confounders. Although SMI showed modest positive correlations with HGS in both hands, this relationship was not retained in multivariable analyses, indicating that the association between muscle mass and functional strength is influenced by additional factors such as age and muscle distribution. Importantly, HGS did not exhibit a significant association with hospitalization length, suggesting that functional measures of muscle strength may not capture the aspects of physiological reserve that are most relevant to postoperative recovery. These findings highlight a clinically meaningful distinction between structural and functional components of sarcopenia, where SMI appears to provide more robust prognostic information in the surgical oncology setting. While HGS remains a simple, non-invasive, and accessible bedside measure, its role should be considered complementary rather than substitutive, particularly in contexts where precise risk stratification is required. These results support the use of CT-derived SMI as a reliable imaging-based biomarker for preoperative assessment, while emphasizing the need for integrated evaluation of muscle status to better inform clinical decision-making.

This study evaluated SMI as a clinically relevant predictor of surgical outcomes and demonstrated that it was independently associated with hospitalization duration after adjustment for key clinical and demographic variables. SMI is readily obtainable from routine preoperative CT imaging without additional procedures or radiation exposure, with the L3 vertebral level serving as a standardized and reliable anatomical landmark [41]. When normalized for height, it provides a comparable measure of sarcopenia across patients [15]. Notably, SMI offered more meaningful prognostic information than conventional indicators such as age and BMI [42, 43], supporting its role as a non-invasive biomarker for identifying high-risk patients who may benefit from closer perioperative monitoring and targeted nutritional or rehabilitation strategies [44, 45]. This is particularly relevant in overweight patients, in whom BMI may obscure underlying muscle depletion, whereas SMI enables more accurate detection of sarcopenia [46–48]. Accordingly, early assessment of muscle quantity using CT-derived measures should be considered as part of routine preoperative evaluation, especially in this subgroup.

SMI also provides insight into the relationship between structural muscle reserves and functional capacity [49–51]. In this study, SMI showed modest correlations with HGS, indicating that muscle mass and functional strength represent related but distinct aspects of sarcopenia [24, 52]. Although HGS demonstrated significant crude associations, it did not retain independent significance after adjustment, suggesting that it may be influenced by transient clinical factors such as inflammation [53], fatigue [54], and patient effort [55]. In contrast, CT-derived measures, including SMI and psoas muscle diameter, reflect structural muscle status and appear more stable in multivariable models. Therefore, HGS should be considered a complementary functional tool rather than a substitute for imaging-based muscle assessment. The slightly stronger association observed for the left hand may suggest that non-dominant measurements better capture underlying functional decline, although this finding warrants further investigation.

In line with our findings, recent studies have emphasized the added value of integrating sarcopenia with systemic inflammatory markers in predictive modeling. For example, Uyar et al. demonstrated that combining sarcopenia with inflammatory indices such as the C-reactive protein–to–albumin ratio (CAR) and the systemic immune-inflammation index (SII) significantly improves prognostic accuracy in patients with gastrointestinal malignancies compared with single-parameter models [56]. These findings support the concept that multi-dimensional and artificial intelligence (AI)-enhanced approaches may provide superior risk stratification compared with isolated measures of muscle mass or function alone. Although the present study focused on structural (SMI) and functional (HGS) muscle parameters, future research should explore the integration of inflammatory and metabolic biomarkers to develop more comprehensive and clinically applicable predictive models.

An additional finding of this study is that psoas muscle diameter remained independently associated with HGS in the adjusted models, whereas SMI did not. This suggests that psoas-based measures may, in some settings, reflect functional muscle performance more directly than broader indices of total muscle mass. However, this should not be interpreted as evidence that psoas measurements can replace SMI. SMI remains the more comprehensive indicator because it is derived from total skeletal muscle area at the L3 level rather than a single muscle group. Consistent with this distinction, a large prospective multicenter study reported only moderate correlation and limited diagnostic agreement between psoas muscle index and total SMI, indicating that psoas-based metrics are not fully representative of whole-muscle-area assessment for cancer sarcopenia [57]. At the same time, both SMI- and psoas-based CT measures have been associated with adverse postoperative outcomes, supporting their complementary rather than interchangeable roles [44]. This is clinically relevant because psoas-derived measures are simpler to obtain in routine practice and have been linked to postoperative complications, mortality, and risk stratification across different surgical settings [58–61]. Their functional relevance is further supported by studies demonstrating associations between psoas-derived CT metrics and HGS across different clinical populations, including cardiovascular surgery and hemodialysis cohorts [62–64]. These findings suggest that psoas diameter may serve as a practical function-oriented surrogate when rapid assessment is needed, whereas SMI remains the more comprehensive marker of overall sarcopenia burden. This may explain why psoas diameter was more strongly related to HGS in our study, while SMI remained more relevant to hospitalization outcomes.

This study has several limitations. Although designed as a prospective observational cohort, its non-randomized nature precludes causal inference between SMI and postoperative outcomes; however, the prospective collection of exposure (preoperative SMI and HGS) and outcome (postoperative hospitalization length) data supports appropriate temporal assessment of associations. The primary outcome, length of stay, may also be influenced by non-clinical factors such as discharge policies, insurance delays, bed availability, and patients’ social circumstances [65], which were not captured in the dataset and may introduce unmeasured confounding. In addition, missing data in key variables, particularly SMI, HGS, and imaging-derived measures, primarily due to incomplete availability of imaging or functional assessments rather than outcome-related factors, resulted in reduced sample size for correlation analyses; the high proportion of missing SMI data was primarily attributable to incomplete availability of Digital Imaging and Communications in Medicine (DICOM) imaging files, inadequate CT scan coverage at the L3 vertebral level, and limited capacity for image segmentation and analysis during the study period; these constraints were logistical rather than clinically driven and were not related to patient outcomes. These analyses were based on unadjusted pairwise associations and were intended as exploratory descriptive assessments rather than tools for clinical inference; therefore, they should not be interpreted as indicative of independent or causal relationships, which were instead evaluated using multivariable models. Comparison of included and excluded patients showed broadly similar baseline characteristics, suggesting that major selection bias is unlikely, although it cannot be fully excluded. In addition, because multivariable analyses were restricted to complete-case datasets, the effective sample sizes differed across models, and the primary findings are therefore based on a subset of the overall cohort. However, it is important to note that the effective sample sizes for the main multivariable analyses were consistent with, or close to, the minimum required sample size determined a priori (*n* = 65), supporting the statistical adequacy of the models despite missing data. Several clinically important variables, including tumor stage, detailed surgical factors (such as procedure duration), postoperative complications, and consistent classification of surgery as elective or emergency, were not available and could not be incorporated into the adjusted models. We also acknowledge that grouping surgical procedures based on anatomical categories may not fully eliminate clinical heterogeneity, as procedures within the same group can differ markedly in physiological impact and expected length of stay. Although surgical procedure category and surgical complexity were used to partially account for operative burden, residual confounding related to surgical urgency and procedural heterogeneity cannot be excluded; moreover, surgical complexity could not be fully modeled in sensitivity analyses due to the absence of low-complexity cases in the complete-case dataset, although comparisons between high and intermediate complexity did not materially alter effect estimates, supporting the robustness of the findings. Importantly, the inclusion of surgical complexity in sensitivity analyses did not materially change the association between SMI and hospitalization length, further confirming the stability of the primary results. Similarly, surgical approach (minimally invasive versus open), an important determinant of postoperative recovery, could not be included due to limited availability and insufficient variability after complete-case restriction, representing an additional potential source of residual confounding. The single-center design may limit generalizability, although inclusion of a heterogeneous population of gastrointestinal malignancies partially enhances applicability. Exclusion of patients with missing key measurements, while necessary for data integrity and accurate SMI estimation, may have introduced selection bias. Furthermore, although established international cutoffs were applied, the lack of universally standardized thresholds for sarcopenia may influence interpretation. Potential variability in CT-based SMI measurement was minimized through blinded assessment by two independent physicians using standardized protocols. Functional assessment was limited to HGS, and other performance measures such as gait speed or muscle endurance were not available. Finally, despite adjustment for major confounders, residual confounding remains possible, and the focus on short-term hospitalization outcomes without long-term follow-up limits broader prognostic interpretation. Despite these limitations, the study provides a methodologically robust and clinically relevant evaluation of the role of sarcopenia in postoperative outcomes among patients with gastrointestinal malignancies and offers a valuable foundation for future multicenter and longitudinal research.

Our findings highlight the potential clinical relevance of preoperative SMI as a marker for identifying patients at higher risk of prolonged hospitalization in patients with GI malignancies, while also underscoring the importance of early identification of sarcopenia as a potentially modifiable condition. Existing evidence suggests that sarcopenia may be partially improved through multimodal approaches, including nutritional optimization and structured exercise programs, which have been associated with favorable effects on muscle mass, strength, and functional capacity [66–70]. Although the present study was not designed to evaluate intervention effects and was limited by its single-center design without external validation, our results support the rationale for further investigation in this area. Future studies should aim to establish standardized and clinically applicable strategies for sarcopenia management, including targeted nutritional, physical, and pharmacological interventions to mitigate treatment-related muscle loss. In addition, the development of validated and widely applicable SMI cut-off values may enhance its utility in routine clinical practice and improve perioperative risk assessment and decision-making.

In conclusion, preoperative SMI appears to be a clinically meaningful imaging-based marker of postoperative recovery in patients with gastrointestinal tumors and may contribute to perioperative risk stratification by identifying patients at greater risk of prolonged hospitalization. Within the framework of this study, HGS was not analyzed as a primary predictor of hospitalization length, but rather as a functional measure of muscle status to determine whether a simple bedside assessment reflects the same underlying physiological domain captured by CT-based muscle quantification. Our findings suggest that although HGS is related to muscle status and selected clinical factors, it does not provide the same prognostic information as SMI and therefore should not be considered a substitute for imaging-based assessment. Instead, these measures appear to offer distinct but complementary information, with SMI better reflecting structural muscle reserve relevant to postoperative recovery and HGS providing an accessible functional perspective on patient status. Accordingly, integrating both structural and functional muscle assessment may improve the comprehensiveness of preoperative evaluation in gastrointestinal oncology, while further multicenter studies with broader clinical endpoints are needed to confirm these findings and define their practical role in routine care. 

## Supplementary Information

Supplementary Table S1. Association of SMI with hospitalization length using continuous and threshold-based definitions (NB models). Supplementary Table S2. Sensitivity analysis of predictors of hospitalization length with and without adjustment for surgical complexity using multivariable NB models. Supplementary Table S3. Spearman’s rank correlation coefficients between SMI, clinical, and functional variables. Supplementary Table S4. Comparison of quantitative baseline characteristics between patients included in and excluded from the correlation analysis. Supplementary Table S5. Comparison of categorical baseline characteristics between patients included in and excluded from the correlation analysis. Supplementary Table S6. Analysis populations and variable-level missingness across models. Supplementary Table S7. Bootstrap-based assessment of model coefficient stability (1,000 resamples). Supplementary Figure S1. Study flow diagram illustrating cohort construction and analysis-specific data availability.

## Data Availability

The data analyzed during the current study are available from the corresponding author upon reasonable request. Please contact Dr. Amirhassan Rabbani at amirhassanrabbani92@gmail.com to obtain access.
