# Supplementary material for: Skeletal muscle index and hand grip strength as predictors of postoperative recovery in patients with gastrointestinal tumors
Source: BMC Gastroenterol. 2026 Apr 23;26:345. doi: 10.1186/s12876-026-04850-1 (PMC13237978; doi:10.1186/s12876-026-04850-1)
Supplement: Supplementary file 1 — Supplementary Table S1. Association of SMI with hospitalization length using continuous and threshold-based definitions (NB models). Supplementary Table S2. Sensitivity analysis of predictors of hospitalization length with and without adjustment for surgical complexity using multivariable NB models. Supplementary Table S3. Spearman’s rank correlation coefficients between SMI, clinical, and functional variables. Supplementary Table S4. Comparison of quantitative baseline characteristics between patients included in and excluded from the correlation analysis. Supplementary Table S5. Comparison of categorical baseline characteristics between patients included in and excluded from the correlation analysis. Supplementary Table S6. Analysis populations and variable-level missingness across models. Supplementary Table S7. Bootstrap-based assessment of model coefficient stability (1,000 resamples). Supplementary Figure S1. Study flow diagram illustrating cohort construction and analysis-specific data availability. [file 12876_2026_4850_MOESM1_ESM.docx]

**Supplementary File**

**Skeletal Muscle Index and Hand Grip Strength as Predictors of Postoperative Recovery in Patients with Gastrointestinal Tumors**

**Supplementary Table S1**. Association of SMI with hospitalization length using continuous and threshold-based definitions (NB models)

| **Exposure Definition** | **Model** | **Variable** | **IRR (95% CI)** | **P-value** |
| --- | --- | --- | --- | --- |
| **SMI (Continuous)** | Univariate | SMI (per 1 cm²/m² increase) | 0.97 (0.95–0.98) | <0.001 |
|  | Multivariable† | SMI (per 1 cm²/m² increase) | 0.94 (0.92–0.97) | <0.001 |
|  | Multivariable† | Psoas muscle diameter | 1.01 (1.00–1.02) | 0.032 |
|  | Multivariable† | Charlson-like category (1 vs 0) | 1.25 (0.98–1.58) | 0.069 |
|  | Multivariable† | Cancer type (Pancreatic vs Hepatobiliary) | 1.21 (0.84–1.76) | 0.306 |
|  | Multivariable† | Cancer type (GI vs Hepatobiliary) | 1.01 (0.54–1.97) | 0.974 |
|  | Multivariable† | Surgical procedure (GI vs reference) | 1.04 (0.77–1.39) | 0.814 |
|  | Multivariable† | Surgical procedure (HPB vs reference) | 0.88 (0.43–1.88) | 0.738 |
| **Sarcopenia (Main cut-off)** | Univariate | Sarcopenia (Yes vs No) | 1.36 (1.06–1.74) | 0.015 |
|  | Multivariable† | Sarcopenia (Yes vs No) | 1.35 (1.07–1.71) | 0.014 |
|  | Multivariable† | Psoas muscle diameter | 0.99 (0.99–1.00) | 0.023 |
|  | Multivariable† | Charlson-like category (1 vs 0) | 1.19 (0.93–1.54) | 0.164 |
|  | Multivariable† | Cancer type (Pancreatic vs Hepatobiliary) | 1.30 (0.89–1.92) | 0.185 |
|  | Multivariable† | Cancer type (GI vs Hepatobiliary) | 0.73 (0.38–1.45) | 0.365 |
|  | Multivariable† | Surgical procedure (GI vs reference) | 1.01 (0.73–1.38) | 0.962 |
|  | Multivariable† | Surgical procedure (HPB vs reference) | 0.61 (0.29–1.31) | 0.199 |
| **Sarcopenia (Alternative cut-off)** | Univariate | Sarcopenia (Yes vs No) | 1.49 (1.17–1.89) | 0.001 |
|  | Multivariable† | Sarcopenia (Yes vs No) | 1.44 (1.14–1.82) | 0.003 |
|  | Multivariable† | Psoas muscle diameter | 1.00 (0.99–1.00) | 0.111 |
|  | Multivariable† | Charlson-like category (1 vs 0) | 1.25 (0.97–1.60) | 0.079 |
|  | Multivariable† | Cancer type (Pancreatic vs Hepatobiliary) | 1.34 (0.93–1.95) | 0.129 |
|  | Multivariable† | Cancer type (GI vs Hepatobiliary) | 0.82 (0.43–1.59) | 0.543 |
|  | Multivariable† | Surgical procedure (GI vs reference) | 0.94 (0.68–1.28) | 0.680 |
|  | Multivariable† | Surgical procedure (HPB vs reference) | 0.63 (0.30–1.32) | 0.212 |

Note: †Multivariable negative binomial regression models were constructed using a screening-based approach. Variables with P < 0.10 in univariate negative binomial analyses were considered for inclusion in the multivariable models. For categorical variables, if at least one level met this threshold, the entire variable was retained. Accordingly, the set of covariates included in each multivariable model may vary depending on the exposure definition and data availability. Due to significant over-dispersion in Poisson models, negative binomial regression was used for all analyses. Effect estimates are presented as incidence rate ratios (IRRs) with 95% confidence intervals (CIs). Continuous SMI was modeled per 1 cm²/m² increase. Two threshold-based definitions of sarcopenia were evaluated based on the exact cut-offs implemented in the analysis code: the primary definition defined sarcopenia as SMI < 55 cm²/m² for males and < 39 cm²/m² for females, while the alternative definition used lower thresholds of SMI < 49 cm²/m² for males and < 31 cm²/m² for females. Reference categories were defined as non-sarcopenia for threshold-based models, hepatobiliary malignancies for cancer type, general and emergency procedures for surgical procedure, and Charlson-like category 0 for comorbidity burden. The consistency of findings across continuous and threshold-based specifications supports a robust association between reduced muscle mass and prolonged hospitalization. SMI used in both continuous and threshold-based models was derived from total skeletal muscle area at the L3 level, including the psoas, paraspinal, and abdominal wall muscles. Multicollinearity was assessed using variance inflation factors (VIF) and adjusted generalized variance inflation factors (GVIF^(1/(2×Df))), with all values below 5, indicating no evidence of problematic collinearity. **Abbreviations:** IRR, incidence rate ratio; CI, confidence interval; SMI, skeletal muscle index; BMI, body mass index; NB, negative binomial; HGS, hand grip strength.

**Supplementary Table S2**. Sensitivity analysis of predictors of hospitalization length with and without adjustment for surgical complexity using multivariable NB models

| **Variable** | **No Complexity Model** | | | **With Surgical Complexity Model** | | |
| --- | --- | --- | --- | --- | --- | --- |
|  | **IRR** | **95% CI** | **P-value** | **IRR** | **95% CI** | **P-value** |
| Intercept | 23.66 | 11.04 – 50.60 | <0.001 | 23.6 | 10.83 – 51.38 | <0.001 |
| Cancer type (Pancreatic & periampullary vs hepatobiliary) | 1.17 | 0.840 – 1.636 | 0.361 | 1.168 | 0.834 – 1.644 | 0.372 |
| Cancer type (Gastrointestinal vs hepatobiliary) | 1.126 | 0.843 – 1.518 | 0.431 | 1.127 | 0.842 – 1.520 | 0.431 |
| Psoas muscle diameter | 1.011 | 1.002 – 1.019 | 0.013 | 1.011 | 1.002 – 1.019 | 0.014 |
| Charlson-like category (1 vs 0) | 1.264 | 1.001 – 1.594 | 0.048 | 1.265 | 1.001 – 1.595 | 0.048 |
| Charlson-like category (2 vs 0) | 1.157 | 0.765 – 1.711 | 0.476 | 1.157 | 0.764 – 1.717 | 0.477 |
| Surgical complexity (High vs Intermediate) * | — | — | — | 1.004 | 0.782 – 1.296 | 0.974 |
| SMI (per 1 cm²/m² increase) | 0.941 | 0.915 – 0.968 | <0.001 | 0.940 | 0.914 – 0.967 | <0.001 |

Note: Results are based on screened multivariable negative binomial regression models constructed using a screening-based approach. Variables with P < 0.10 in univariate negative binomial analyses were considered for inclusion in the multivariable models. For categorical variables, if at least one level met this threshold, the entire variable was retained. The “No Complexity Model” and the “With Surgical Complexity Model” share the same set of screened covariates, with the latter additionally including surgical complexity (High vs Intermediate) to assess the robustness of the associations. The asterisk (*) indicates that surgical complexity was modeled as High vs Intermediate because the Low complexity category was not represented in the complete-case multivariable dataset. Effect estimates for the remaining variables were derived under the same screening framework and showed minimal changes after inclusion of surgical complexity, supporting the robustness of the findings. Surgical approach was not included in these models due to insufficient completeness and variability in the complete-case dataset. SMI was defined based on total skeletal muscle area at the L3 level and analyzed independently from psoas muscle diameter. Multicollinearity was assessed using variance inflation factors (VIF) and adjusted generalized variance inflation factors (GVIF^(1/(2×Df))), with all values below 5, indicating no evidence of problematic collinearity. **Abbreviation:**  IRR, incidence rate ratio; CI, confidence interval; NB, negative binomial; SMI, skeletal muscle index.

**Supplementary Table S3**. Spearman’s rank correlation coefficients between SMI, clinical, and functional variables

| **Variable 1** | **Variable 2** | **rs (95% CI)** | **P-value** |
| --- | --- | --- | --- |
| SMI | LoH | -0.49 (-0.67, -0.25) | <0.001 |
| SMI | Age | -0.15 (-0.41, 0.12) | 0.263 |
| SMI | BMI | 0.21 (-0.06, 0.46) | 0.115 |
| SMI | HGS - Right | 0.27 (0.00, 0.50) | 0.047 |
| SMI | HGS - Left | 0.29 (0.02, 0.52) | 0.033 |
| LoH | Age | -0.14 (-0.39, 0.14) | 0.309 |
| LoH | BMI | -0.31 (-0.53, -0.04) | 0.021 |
| LoH | HGS - Right | -0.04 (-0.31, 0.23) | 0.773 |
| LoH | HGS - Left | -0.16 (-0.41, 0.11) | 0.230 |
| Age | BMI | -0.03 (-0.30, 0.24) | 0.820 |
| Age | HGS - Right | -0.43 (-0.63, -0.18) | <0.001 |
| Age | HGS - Left | -0.38 (-0.59, -0.12) | 0.004 |
| BMI | HGS - Right | -0.12 (-0.38, 0.16) | 0.379 |
| BMI | HGS - Left | -0.05 (-0.31, 0.23) | 0.742 |
| HGS - Right | HGS - Left | 0.90 (0.84, 0.94) | <0.001 |

Note: Spearman’s correlation coefficients (rs) with 95% confidence intervals (CI) and P-values were used to assess associations among skeletal muscle index (SMI), hospitalization length (LoH), age, body mass index (BMI), and hand grip strength (HGS) for both hands. Standard errors were estimated using the formula proposed by Fieller, Hartley, and Pearson. After removing missing data, 56 valid cases were included in the analysis. SMI represents total skeletal muscle area at the L3 level and is distinct from psoas muscle measurements. **Abbreviations:** SMI, Skeletal Muscle Index; LoH, Length of Hospitalization; BMI, Body Mass Index; HGS, Hand Grip Strength; CI, Confidence Interval.

**Supplementary Table S4**. Comparison of quantitative baseline characteristics between patients included in and excluded from the correlation analysis

| **Variable** | **Group** | **N** | **Mean ± SD** | **Median (Q1–Q3)** |
| --- | --- | --- | --- | --- |
| Age (years) | Included | 56 | 58.23 ± 11.34 | 58.5 (50–66.25) |
|  | Excluded | 176 | 59.42 ± 13.18 | 59.5 (50.75–68.25) |
| BMI (kg/m²) | Included | 56 | 25.83 ± 5.79 | 25.45 (21.25–29.56) |
|  | Excluded | 114 | 24.42 ± 4.97 | 24.0 (21–28.28) |
| Psoas muscle diameter | Included | 56 | 124.69 ± 22.60 | 121.7 (104.18–142.98) |
|  | Excluded | 64 | 120.27 ± 31.01 | 115.6 (99.72–136.43) |
| SMI (cm²/m²) | Included | 56 | 46.49 ± 6.95 | 46.67 (41.70–50.49) |
|  | Excluded | 18 | 42.59 ± 10.45 | 40.97 (37.74–47.83) |
| Hospitalization length (days) | Included | 56 | 6.61 ± 3.46 | 6 (4–8.25) |
|  | Excluded | 80 | 6.09 ± 3.21 | 5.5 (4–7.25) |

Note: Continuous variables are presented as mean ± standard deviation (SD) and median (interquartile range, Q1–Q3). The “Included” group represents patients with complete data available for all variables required for the correlation analysis, while the “Excluded” group includes patients with missing data in at least one relevant variable (e.g., SMI, HGS, or psoas muscle measurements). This comparison was performed to assess potential selection bias resulting from complete-case analysis. SMI values were derived from total skeletal muscle area at the L3 level. **Abbreviations**: BMI, body mass index; SMI, skeletal muscle index; SD, standard deviation.

**Supplementary Table S5**. Comparison of categorical baseline characteristics between patients included in and excluded from the correlation analysis

| **Variable** | **Level** | **Included n (%)** | **Excluded n (%)** |
| --- | --- | --- | --- |
| Sex | Female | 23 (41.1) | 78 (44.1) |
|  | Male | 33 (58.9) | 99 (55.9) |
| Cancer type | Gastrointestinal | 30 (53.6) | 64 (36.2) |
|  | Hepatobiliary | 12 (21.4) | 59 (33.3) |
|  | Pancreatic/Periampullary | 14 (25.0) | 54 (30.5) |
| Charlson-like category | 0 | 34 (60.7) | 127 (71.8) |
|  | 1 | 18 (32.1) | 34 (19.2) |
|  | ≥2 | 4 (7.1) | 16 (9.0) |
| Surgical procedure | HPB | 24 (42.9) | 108 (61.0) |
|  | GI | 14 (25.0) | 26 (14.7) |
|  | Upper GI | 18 (32.1) | 28 (15.8) |

Note: Categorical variables are presented as frequency (percentage). The “Included” group consists of patients with complete data available for correlation analysis, whereas the “Excluded” group includes those with missing values in one or more variables. This comparison was conducted to evaluate potential selection bias associated with missing data. Percentages are calculated within each group. Abbreviations: HPB, hepatopancreatobiliary; GI, gastrointestinal.

**Supplementary Table S6**. Analysis populations and variable-level missingness across models

| **Analysis** | **Outcome** | **Variables required** | **Effective N** | **Key variables with missing data (% missing)** |
| --- | --- | --- | --- | --- |
| Full study cohort | — | Cohort inclusion criteria | 233 | — |
| Patients with hospitalization length observed | Hospitalization length | Hospitalization length | 136 | Hospitalization length (41.63%) |
| Patients with SMI observed | Skeletal Muscle Index (SMI) | SMI | 74 | SMI (68.24%) |
| Patients with HGS-right observed | Hand Grip Strength (Right) | HGS (right hand) | 169 | HGS-right (27.47%) |
| Patients with HGS-left observed | Hand Grip Strength (Left) | HGS (left hand) | 169 | HGS-left (27.47%) |
| Screened multivariable LOS model (complete-case) | Hospitalization length | Hospitalization length, Cancer type, Psoas muscle diameter, Charlson-like category, Surgical procedure, SMI | 64 | SMI (68.24%), Psoas muscle diameter (48.50%), Hospitalization length (41.63%) |
| Multivariable HGS-right model (complete-case) | Hand Grip Strength (Right) | HGS (right), Age, BMI, Sex, Cancer type, Psoas muscle diameter, Charlson-like category, Surgical procedure, SMI | 64 | SMI (68.24%), Psoas muscle diameter (48.50%), BMI (27.04%), HGS-right (27.47%) |
| Multivariable HGS-left model (complete-case) | Hand Grip Strength (Left) | HGS (left), Age, BMI, Sex, Cancer type, Psoas muscle diameter, Charlson-like category, Surgical procedure, SMI | 64 | SMI (68.24%), Psoas muscle diameter (48.50%), BMI (27.04%), HGS-left (27.47%) |
| Correlation analysis (complete-case) | Correlation analysis | Age, BMI, HGS (right), HGS (left), SMI, Hospitalization length | 56 | SMI (68.24%), Hospitalization length (41.63%), BMI (27.04%), HGS (27.47%) |

Note: Effective sample sizes are based on complete-case analyses for each model. Missing data occurred primarily at the variable level due to incomplete availability of imaging-derived measurements (e.g., skeletal muscle index and psoas muscle diameter) and functional assessments (hand grip strength), rather than exclusion at the cohort level. Percentages represent the proportion of missing observations relative to the total cohort (n = 233).

**Supplementary Table S7**. Bootstrap-based assessment of model coefficient stability (1,000 resamples)

| **Variable** | **Mean** | **Exp (Mean)** | **SD** | **Median** | **2.5th Percentile** | **97.5th Percentile** | **Direction Consistency (%)** |
| --- | --- | --- | --- | --- | --- | --- | --- |
| (Intercept) | 4.46 | 86.49 | 1.19 | 4.57 | 1.79 | 6.45 | 100 |
| Age | -0.012 | 0.99 | 0.008 | -0.013 | -0.028 | 0.003 | 93.8 |
| BMI | -0.008 | 0.99 | 0.019 | -0.006 | -0.053 | 0.026 | 62.4 |
| Sex (Male) | 0.158 | 1.17 | 0.24 | 0.154 | -0.343 | 0.614 | 75.8 |
| Cancer type (Pancreatic/periampullary) | 0.281 | 1.32 | 0.378 | 0.259 | -0.39 | 0.999 | 74.9 |
| Cancer type (Gastrointestinal) | -0.166 | 0.85 | 0.725 | -0.314 | -1.33 | 1.25 | 66.8 |
| Psoas muscle diameter | -0.001 | 1.00 | 0.009 | -0.001 | -0.02 | 0.015 | 56 |
| HGS (Right) | 0.018 | 1.02 | 0.025 | 0.019 | -0.03 | 0.066 | 77.6 |
| HGS (Left) | -0.025 | 0.98 | 0.024 | -0.025 | -0.078 | 0.023 | 86.7 |
| Charlson-like category (1 vs 0) | 0.052 | 1.05 | 0.152 | 0.057 | -0.274 | 0.311 | 66.1 |
| Charlson-like category (2 vs 0) | 0.123 | 1.13 | 0.252 | 0.132 | -0.4 | 0.581 | 72.3 |
| Surgical procedure (GI vs ref) | 0.011 | 1.01 | 0.187 | 0.002 | -0.325 | 0.408 | 50.2 |
| Surgical procedure (HPB vs ref) | -0.384 | 0.68 | 0.839 | -0.419 | -1.85 | 1.3 | 68 |
| **SMI** | **-0.029** | 0.97 | **0.027** | **-0.031** | **-0.08** | **0.028** | **88.2** |

Note: Bootstrap resampling (1,000 iterations) was used to assess model stability. Results are based on 566 successful iterations with complete coefficient estimation. Direction consistency represents the proportion of bootstrap samples in which the coefficient retained the same sign (positive or negative) as the original model. The Exp (Mean) column represents the exponentiated coefficients, providing an interpretable measure analogous to incidence rate ratios (IRRs).


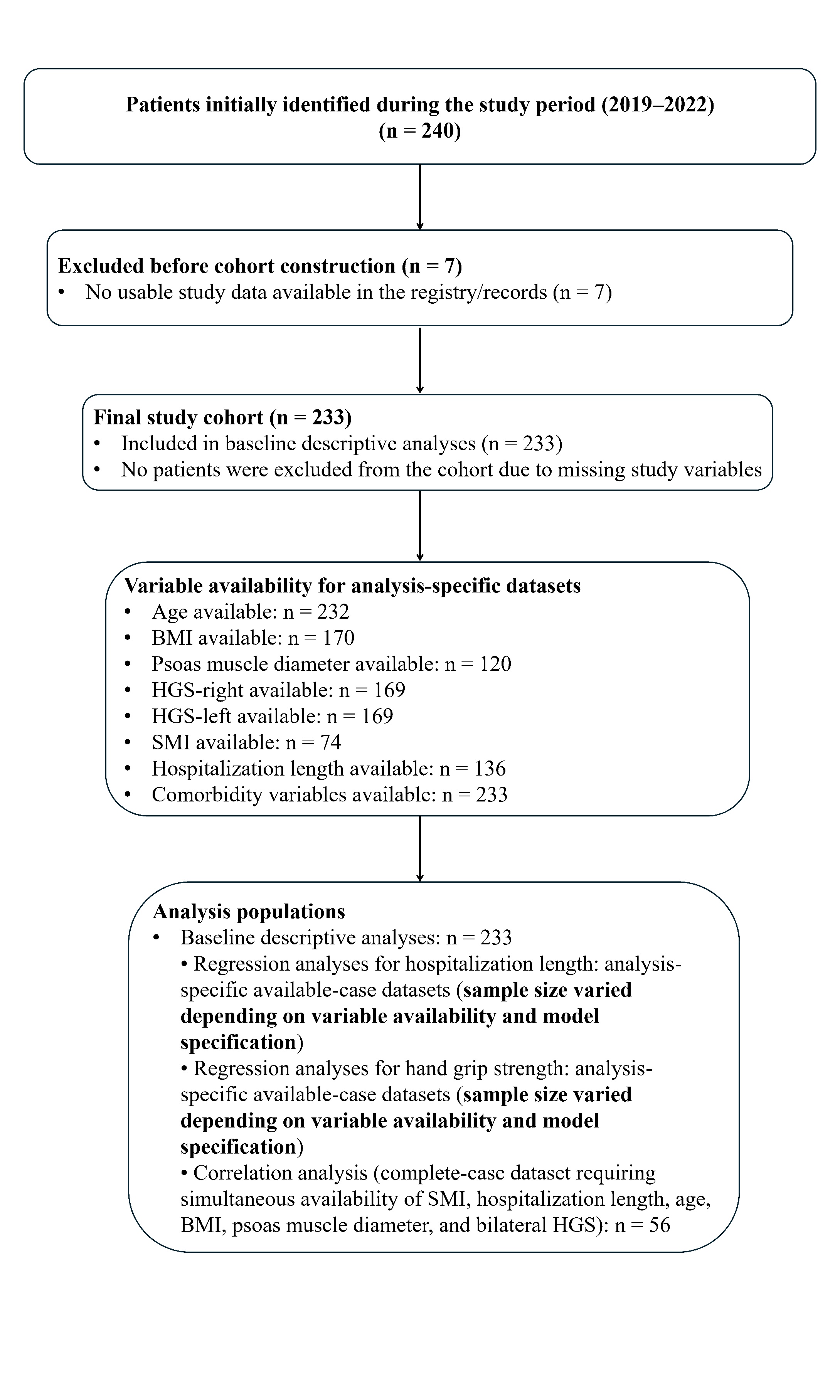


Supplementary Figure S1. Study flow diagram illustrating cohort construction and analysis-specific data availability [*Study flow diagram showing cohort construction and analysis-specific data availability. Of 240 patients identified during the study period, 7 were excluded before cohort construction because no usable study data were available. The final cohort consisted of 233 patients and was used for baseline descriptive analyses. Missing data were handled at the variable level; therefore, patients were not excluded from the study cohort solely due to the unavailability of one or more variables. Instead, analysis-specific datasets were constructed using available-case or complete-case approaches depending on the requirements of each analysis, rather than excluding patients at the cohort level.* *Multicollinearity was assessed in all regression models using variance inflation factors, with no evidence of problematic collinearity.*]
